# Perceived benefits and negative consequences of alcohol consumption in women living with HIV: a qualitative study

**DOI:** 10.1186/s12889-016-2928-x

**Published:** 2016-03-15

**Authors:** Robert L. Cook, Christa L. Cook, Manju Karki, Kathleen M. Weber, Kathleen A. Thoma, Chelsea M. Loy, Lakshmi Goparaju, Bridgett Rahim-Williams

**Affiliations:** Departments of Epidemiology and Medicine, University of Florida, 2004 Mowry Road, Gainesville, FL 32610 USA; Department of Family, Community, and Health System Science, University of Florida College of Nursing, PO Box 100197, Gainesville, FL 32610-0197 USA; Department of Pathology, Immunology and Laboratory Medicine, University of Florida, Gainesville, FL 32610 USA; Cook County Health and Hospital System and Hektoen Institute of Medicine, 2225 W Harrison St, Chicago, IL 60612 USA; Clinical Research Specialist, UF CARES, University of Florida Center for HIV/AIDS, Research, Education & Service, 653-1 West 8th Street, LRC 3rd Floor L-13, Jacksonville, FL 32209 USA; Women’s Interagency HIV Study (WIHS), Georgetown University Medical Center, 2115 Wisconsin Ave NW, Suite 130, Washington DC, 20007 USA; Department of Public Health, Bethune-Cookman University, College of Health Sciences, 640 Dr. Mary McLeod Bethune Blvd., Daytona Beach, Florida, 32114 USA

## Abstract

**Background:**

Women living with HIV have increased prevalence of medical and psychological comorbidities that could be adversely affected by alcohol consumption. Little is known about their unique motivations for drinking or perceptions of HIV-related consequences. In preparation for an alcohol intervention study, we sought to better understand reasons for drinking and perceived consequences of alcohol consumption among a sample of women living with HIV.

**Methods:**

Four focus groups, with a total of 24 adult women (96 % African-American, 88 % HIV-positive), were conducted in Jacksonville, FL, Washington, DC and Chicago, IL. Focus group discussions were tape-recorded and transcribed verbatim; a conventional content analysis approach was used to identify themes, that were then grouped according to a biopsychosocial model.

**Results:**

Regarding reasons for drinking, women described themes that included biological (addiction, to manage pain), psychological (coping, to escape bad experiences, to feel in control), and social (peer pressure, family). Themes related to consequences from alcohol included biological (damage to body, poor adherence to medications), psychological (risky or regrettable behavior, memory loss), and social (jail, loss of respect, poor choices). When discussing how their drinking impacted their health, women focused on broader issues, rather than HIV-specific issues.

**Conclusion:**

Many women living with HIV are drinking alcohol in order to self-manage pain or emotions, and their perceived consequences from drinking extend beyond HIV-specific medical issues. Most participants described themes related to psychological issues and situations that are common in women living with HIV. Interventions to address drinking should inquire more specifically about drinking to manage pain or emotion, and help women to recognize the potential adverse impact of alcohol on comorbid health issues, including their own HIV infection.

## Background

HIV infection remains a prominent issue for women in the United States. In 2014, the Centers for Disease Control and Prevention reported that one in five people living with HIV (PLWH) are female [1]. In the U.S., rates of HIV infection in women are substantially higher among African-American and Hispanic women, and women living with HIV often have previous exposure to stressors such as poverty and violence [2]. With the introduction of highly-active antiretroviral therapy (ART) in the 1990s, HIV can be a chronic disease for most persons living with HIV, but there are gender disparities related to HIV care engagement and HIV viral suppression [2–4]. Therefore, we need to understand barriers to successful HIV health outcomes that are currently relevant to women living with HIV.

Hazardous alcohol consumption is adversely associated with several relevant HIV health behaviors and outcomes, including non-adherence to ART, risky sexual behavior, HIV disease progression, liver disease, and earlier death [5–10]. Alcohol consumption is also linked to violence, mental health conditions and symptoms, and traumatic events or stressors, including a positive diagnosis of HIV [11–15]. Most public health officials define “hazardous drinking” for women as a high weekly consumption (more than 7 drinks per week), or consumption of four or more drinks in one sitting (often defined as binge drinking) [16]. Among women living with HIV infection, approximately 10–20 % report current hazardous drinking [5, 7], and many more have a past history of hazardous drinking and are at risk for relapse [17]. However, a focus only on the quantity and frequency of alcohol consumption may not fully capture the range of benefits or harms that women may experience from drinking.

Interventions to reduce hazardous drinking are widely recommended but they do not always address reasons for drinking [18]. Women may drink alcohol for different reasons than men, and they may be more vulnerable to some types of consequences. In general, persons often drink alcohol to cope with symptoms or to participate in social activities [19, 20]. However, little is known about unique reasons for drinking or consequences in women living with HIV. We hypothesized that both the reasons for alcohol consumption and the potential consequences could be considered within the context of the biopsychosocial model of health, which posits that addiction and health behavior can be mapped to biological, psychological, or social aspects of health [21, 22]. In preparation for a clinical trial to reduce drinking in women living with HIV, we sought to ensure that we addressed issues that were salient to this population, including reasons for drinking alcohol and perceived consequences that might improve with alcohol cessation. We were especially interested in whether women would identify specific aspects of HIV health in relation to alcohol consumption.

## Methods

### Study design

We obtained qualitative data using focus groups [23], and used a conventional content analysis approach for data analysis. Conventional content analysis is appropriate when investigators seek to obtain information without imposing preconceived themes [24].

### Study population

We sought to include a range of women affected by alcohol and HIV infection in three locations where we were planning to conduct an alcohol intervention study. We first conducted two focus groups in Chicago in 2007. Focus group participants were recruited from the ongoing Women’s Interagency HIV Study (WIHS) [25], a cohort study of women living with HIV as well as women without HIV who had similar demographic and socioeconomic characteristics. At that time, we did not want to disclose our participants’ HIV status by their participation in focus groups limited to HIV-positive women, so we decided to include a few HIV-negative women within the focus groups to maintain anonymity. The two focus groups included a total of 14 women; all were African-American and 3 were HIV-negative. Neither the focus group interviewer nor the research coders could determine which participants were HIV-positive or negative. In 2009, we chose to conduct focus groups at two additional sites that would be part of a clinical trial, and the site leaders recommended separate groups for HIV-positive women only. In Washington DC, 4 HIV-positive women from the WIHS cohort who reported current drinking participated in one group, and in Jacksonville, FL, 6 HIV-positive women with current or past drinking were recruited from an academic medical center and from members of a local HIV research Community Advisory Board.

### Ethics, consent, and permissions

Prior to data collection, we received approval from Institutional Review Boards at the University of Florida (Jacksonville), Rush University (Chicago), and Georgetown University (Washington DC). Written informed consent was obtained at the Washington DC site, whereas verbal consent was obtained from participants in Jacksonville and Chicago. All participants were informed that the objective of the focus groups was to learn more about alcohol and HIV infection in women.

### Study instrument

We created a list of semi-structured, open-ended questions to enhance discussion (Table 1). The questions sought to identify both benefits and harms related to drinking, reasons for drinking and reasons to stop drinking, consequences of drinking (including sexual consequences), and perceived benefits and harms of alcohol as it relates to HIV infection. By asking about both benefits and harms related to drinking, we sought to understand factors that may influence women’s “decisional balance” when contemplating a change in behavior such as alcohol consumption. After the initial focus groups were held in Chicago, and based on input from an HIV Community Advisory Board in Jacksonville, we added additional prompts to learn more about past drinking experiences, influences of friends and family, to probe more specifically about the connections between drinking, pain, and medication adherence, and to assess interest in an upcoming clinical trial related to drinking (Table 1).

**Table 1:** Focus group open-ended question guide

What are some places where women drink alcohol? Lets talk about each – what are some reasons that women drink in each of these places?
What are some fun or enjoyable situations in which women drink alcohol?
What are some bad things that could happen if women drink alcohol?
When is drinking a problem and when is it not a problem? How can people tell if alcohol is causing any harm?
We would like to get your thought on why you think some women drink too much. Could you share some reasons?
What are some reasons that women might choose to cut down on their drinking?
What are some options that women have to help them cut back or stop drinking?
How is alcohol related to sexual behaviors?
How is alcohol different from other drugs that people might use to get high (especially in terms of its effect on a person). How is it better, how is it worse?
Are there any ways that alcohol is helpful for persons with HIV infection?
Are there any ways that alcohol is harmful for persons with HIV infection?
^a^What role do you think pain has on an individual’s desire or need to drink?
^a^When did you start heavy drinking? Was there an event/factor or other reasons that influenced your drinking?
^a^Some women say their friends and family influence their drinking – what about for you or your friends/family that drink?
^a^Some women report that they take their medications less when they are drinking – why might this happen?
^a^The study we are planning will use a medication to see if it helps women to cut back on their drinking and to be healthier. What do you think will make the study attractive for women to participate?

^a^Questions that were added to the final two focus groups

### Study procedures

Focus groups were conducted by a primary facilitator, who reviewed the consent document, answered questions, reaffirmed that participation was voluntary, and reminded persons not to make any statements that would identify themselves or another individual. In Chicago and Washington DC, information about participants’ demographic characteristics and drinking history was obtained from their most recent WIHS cohort data, whereas in Jacksonville we only had information about race and age. The focus groups were recorded and transcribed by a professional transcriptionist.

### Data analysis

Data from four focus groups including 24 women were included for analysis. Two members of the research team conducted an initial coding analysis of the transcriptions, using an inductive, exploratory approach to identify themes related to reasons for drinking and consequences of drinking [24, 26]. Transcripts were color-coded independently, and the team met weekly to discuss emergent codes/categories and resolve discrepancies. A third researcher independently coded transcripts and identified themes using the same process. The three coders discussed their findings together, and also with an ongoing Qualitative Data Analysis Workgroup, an interdisciplinary team of researchers within and outside of the University of Florida. After this input, the themes were grouped within the context of the biopsychosocial model [21, 22] and exemplary quotes were identified for each theme. Although we could identify different persons’ responses in the transcripts, the focus groups were intended to be as anonymous as possible. Therefore, we did not prospectively plan to link individual participant information to the transcripts and did not maintain any code that would allow us to link specific statements to specific characteristics of individuals. Similarly, we did not attempt to compare themes according to geographic location due to the limited numbers of women from each location.

## Results

Nearly all of the 24 women who participated in the focus groups were African-American (*n* = 23); most had HIV infection (*n* = 21), and their ages ranged from 22–59 years. Themes that encapsulated the reasons that women drink and the perceived consequences of drinking are summarized in Table 2. Exemplary quotations chosen to represent theme are presented in *italics* below. The themes and examples are not presented in any specific order, but are meant to be inclusive of the full range of ideas that were raised by study participants.

**Table 2:** Themes related to reasons for drinking and consequences of drinking in 24 women with HIV Infection

	Reasons for drinking	Consequences of drinking
Biological	• Addiction • To feel better • Manage pain	• Not taking medications • Blackouts • Physical damage to body
Psychological	• To feel in control • Coping – escaping • Low self-esteem • Get through bad experiences • To have courage/confidence	• Changes in behavior • Poor choices and decisions • Sexual risk taking
Social	• Socialize • Influenced by friends • Influenced by family/parents	• Neglect • End up in jail • Loss: respect, job, relationships

## Reasons for drinking

### Biological

Biological reasons for drinking included addiction, positive physical sensations, and drinking to relieve pain. For example, several participants indicated that they could not stop drinking once they started. Some noted that they liked the way they felt when drinking. Drinking in response to pain was mentioned by multiple participants, and the pain could have represented true physical pain, emotional pain, or a combination.

*The next day, I don’t feel good, my stomach acting up, and I’d say Lord, why I drink so much, but then I’d turn around and drink again. And I say Lord I got to stop drinking, but turn around and start drinking again. You know and that’s not a good feeling, you know, knowing that you’re killing yourself.*

*I like feeling drunk. I like feeling high. It’s like it wasn’t no feeling that made me feel any better but to drink. And nothing that nobody could say to me, oh just get over it and you’ll be ok. I didn’t want to hear that.*

*You drink to take the pain away.*

### Psychological

Many women drank in order to feel control over their surroundings, and several noted the use of alcohol to help them feel more courageous. Women also frequently noted use of alcohol as a type of self-management of their mood, to cope with stress or life issues, and to cover up their low self-esteem.

*You know addicts are control freaks even when we’re drunk, we are in control even if we control by not participating. Like for me, a long time ago, when I wanted to control, I would just withdraw, because that meant everybody in the room would be like, what’s wrong with her. Why she not, you know, so I had that sort of control.*

*It’s Dr. Jekyll and Mr. Hyde, that’s what I call it because when you’re not drinkin’ you timid, and then as soon as you start drinkin’, you get more vocal, and then you say the same thing over and over, you understand, you understand, you understand, you see what I mean. You say it over and over again, over and over again. And you know and also that alcohol gives you false courage, it gives you false courage. And then you crash.*

*You can be in denial and can’t face what’s going on, you just want to erase it. You don’t want to think about it. And the bottle just feels like it’s your friend, that’s what you’re coping with. That’s your friend. You don’t want to face reality; you just want to be somewhere else.*

*There are quite a few ladies that I have talked to that stay drunk so I don’t have to deal with this issue and you see them day after day because they have not come to grip with what’s going on about their HIV, or whatever problem it is that they’re going through.*

*I was, there wasn’t much to like about myself. I didn’t like, do you want me to be specific? I didn’t like the circumstances I was in, I didn’t like my living conditions, I didn’t like how I was carrying myself on as a female, I didn’t like, I didn’t like nothing about my existence. My total existence was fairly displeasing to me. And sometimes it (alcohol) can bring me out of my shell sometimes; you know, compensating for all those feelings of inferiority that were pretty much inherent.*

### Social

Many women reported drinking for social reasons, such as being in the company of someone drinking, having fun, or being in places where people were drinking. Family and peer influences were also commonly cited, and some women noted how they started drinking at an early age or adolescence due to family exposures or peer influences.

*We go to bars or at each others’ house to start. Same places; just different scenarios. Just want to party and have fun.*

*Before I started hanging out with my friends, yes. Because I was very anti-drinking. They would drink, and I knew that they drank, but I had martial arts and, I had martial arts 6 days a week, so I was always busy. And sometimes on Sunday I had a tournament, so I wasn’t partying with them, but I had gotten hurt in martial arts, so for about 2 or 3 months I stopped and started to socialize with my friends a little bit more, and went to a party and that’s where I started the drinking.*

*My mom considered herself to be a functioning alcoholic. And I’ve never really seen her too many days when she wasn’t drunk or high, you know, that’s what she did every day. She woke up, she didn’t have a water, she drunk beer like that was what she had. So it was normal for me, I guess, my everyday life.*

## Consequences of drinking

### Biological

Participants identified several biological consequences of drinking, including some more immediate consequences (e.g. black-outs, hangovers, physical assaults). Several women noted that they did not take good care of themselves when drinking. Many women recalled severe consequences of drinking from their past. When discussing how their drinking impacted their health, the participants focused on broad issues rather than HIV-specific issues, unless they were specifically asked about HIV infection.

*Alcohol interferes with their HAART medication and that could be really damaging, it could make them become resistant to medication, it’s not good, it’s not good to gamble.*

*I had drunk so much at one time that I had a pancreatic ulcer I didn’t care about that ulcer I had. I was tripping, I was so sick. It was like, I walked like a square block, each square that I walked past on the block, it seemed like I was gagging and throwing up. I went to the hospital, and the doctor did like that and I about jumped off the bed. He said, you got ulcers, no more drinking for you. And people don’t realize that, it can eat up your liver you know, you just don’t know.*

*You’re really not going to take care of yourself, you know. I mean they’re incompatible and an alcoholic doesn’t take care, of course they can drink and like, you know have a drink once a year. Even a person with HIV can do that, but that person with a problem drinking, problem drug use, if I’m using drugs, everything else is completely out the window. I mean my health, my medication, any responsibility that I have, I don’t even eat. I’m doing good to keep myself hydrated at all. Everything goes.*

*If you have HIV and you drink, don’t drink if you love life, continue to take your medicine. Because that’s double jeopardy, the medicine and the alcohol on your liver, that’s double jeopardy.*

### Psychological

Women noted several psychological consequences of drinking, including effects on emotions, decisions, and behaviors. Specific psychological consequences included out of control behavior, criminal activity, bad choices, and risky sexual behavior. Some women noted that they had different consequences from different types of alcoholic beverages, with liquor having a different effect than beer, for example.

*Because you know you get drunk and you want to go do Sally husband, and Sally don’t play.*

*I stopped for like a year and I started back gradually drinking beer and everything. Now I like to drink a lot of beer, I would drink liquor if somebody pissed me off, but I try to give people fair warning, if I start drinking you ain’t going to like me, that keep people back off me. Let me go ahead and do my thing. Now really, like I said, I just drink beer, that’s every day, every night.*

*I woke up and didn’t know where I was and I was in the bed with a guy that I didn’t know.*

### Social

Women identified numerous social consequences of drinking. For example, some women discussed how drinking aversely affected their relationships with their family or their friends, or how they were later embarrassed about things they had done or said. Also, several women identified social consequences related to breaking the law or societal rules.

*Your job, you neglect your family, you can have your children taken away from you, and all kinds of things.*

*Yes, because it was causing more problems at the time. I lost everything. I lost a job, relationship, respect, respect from family, I lost everything, and at the time, yes, certainly, I would have done it. And even when I went to AA, I’m sitting around people where I’m like, wow, my life isn’t that bad, but even realizing it, it was, I had hit bottom, and that’s where I sat.*

*When I drink, I break out in handcuffs. That’s what happens when I drink…(*When you say ‘break out in handcuffs’ what do you mean?) *I mean I’m pretty much going to do something stupid, and I’m going to end up in jail. I just lose complete control of myself. It’s going to be either other drugs and then I’m going to be in real trouble.*

*But socially, I will drink a lot when I’m you know, like I said, I’ve gotten a couple DWIs, blew a .12 and really I didn’t feel like I was drunk, you know… I almost got a third one, one time, and that time I really was drunk because I was sick, and they didn’t give me a DWI, thank God that time.*

## Discussion

Because alcohol can adversely affect a range of HIV-related health outcomes, many experts suggest better integration of substance abuse interventions into HIV primary care [27]. Suggested interventions include referring persons to substance abuse treatment, prescribing medications, or conducting brief interventions [16]. However, it can be challenging to identify those persons who would most benefit from alcohol intervention and to understand factors that may influence their decisions to change drinking behavior. In this study, women identified reasons and consequences that appear to be especially relevant in the context of the lives of women living with HIV. The data clearly demonstrate the impact of alcohol across biological, behavioral, and social dimensions, consistent with a biopsychosocial model of health.

For example, many women cited alcohol use as a self-management strategy for addressing pain, including both physical and emotional pain. Pain is common among individuals with HIV infection, and persons often drink alcohol to self-manage or mask ongoing physical or psychological pain or distress [28–31]. If women are drinking alcohol to self-manage emotional or physical pain, then interventions to reduce drinking may need to provide alternative strategies to manage the pain. Similarly, many women stated that they drank to address psychological symptoms including low self-esteem, stress and depression. Finally, as noted in other literature [32, 33], social aspects of drinking with family and peers were clearly relevant in terms of current and past drinking behavior. Many women in this study discussed how their family’s drinking behavior influenced their own drinking at a young age.

Regarding drinking consequences, we sought to identify whether participants perceived that alcohol had an effect on any HIV-specific health issues. For the most part, women in this study identified more general and immediate health consequences, or health issues from their past, rather than current consequences specific to HIV infection. Although alcohol consumption can affect HIV medication adherence, HIV disease progression, HIV comorbidities, and increases in viral load [5–10], few of the women participating in our focus groups specifically mentioned these consequences until after being specifically prompted for this information. Because many HIV-related health issues affected by alcohol are not immediate, many women living with HIV may not be aware of the long-term consequences. Therefore, providing women living with HIV with more education and guidance about these issues should be a priority.

Women also noted several behavioral and social consequences of drinking. Links between alcohol consumption and sexual behavior were commonly noted, a connection that is especially relevant to HIV transmission in women [34]. Several women described how alcohol resulted in their becoming involved in sexual activities that they did not intend, while others also acknowledged that alcohol also affected their own personal sexual expectancies and desires. Women often drink for different reasons than men, for example in response to depression [12, 35], or in association with intimate partner violence [14] or other traumatic events or stressors [36].

Few other studies have examined reasons for drinking or drinking consequences in persons with HIV, regardless of gender. One recent study compared reasons for drinking to the amount of alcohol consumption in a HIV clinic population in New York [20]. The investigators found that persons who were drinking to cope or to self-manage problems drank a larger amount of alcohol than persons who drank for other reasons. The investigators did not present the results by gender, indicating they were similar, but fewer than 25 % of the sample was female. Another recent study from the same group found that one third of persons with HIV were unaware of medical risks of drinking [37]. Another qualitative study of women in Africa found that women were motivated to use alcohol to manage their emotions, facilitate social engagement, and to achieve a sense of empowerment even while recognizing the potential consequences of these strategies [38].

Some limitations of the study warrant mention. First, the specific questions in the interview guide varied somewhat across sites, although at each site women were specifically asked about reasons that they drank and to identify perceived consequences of drinking. In order to protect anonymity, we did not set up a system that would allow us to link statements to demographic or health information from specific participants, and therefore could not make comparisons according to individual factors. Similarly, we could not compare responses of women with HIV to the few women without HIV in this study, and therefore we cannot conclude whether or not women with HIV infection have distinct benefits or consequences compared to similar women without HIV infection. The limited number of women from each setting precluded the ability to make comparisons across sites. The interviews were done in preparation for a clinical trial, and thus we did not confirm whether thematic saturation was achieved. Finally, some participants may have been uncomfortable discussing their individual HIV-specific reasons and consequences of drinking within a focus group setting.

## Conclusion

We identified reasons for drinking and consequences of drinking among women living with HIV. Many of the themes are currently relevant, including the use of alcohol to manage pain, emotional symptoms, or to gain confidence. Clinicians and public health personnel who conduct alcohol interventions should inquire about reasons for drinking, because women may be drinking to self-manage another conditions such as pain or depression [35]. Family and social network influences should be considered, as these can also have a strong influence on alcohol intervention motivation or outcomes. General assessments of drinking consequences, such as the Short Inventory of Problems [39], may help women recognize consequences from drinking that could improve from reduction in alcohol consumption. However, women may not always be aware of the potential medical consequences, and more education on HIV-specific medical consequences may be important.

## Abbreviations

HIV: human immunodeficiency virus
PLWH: people living with HIV
ART: antiretroviral therapy
